# HIV Testing Trends Among Persons with Commercial Insurance or Medicaid — United States, 2014–2019

**DOI:** 10.15585/mmwr.mm7025a1

**Published:** 2021-06-25

**Authors:** Kirk D. Henny, Weiming Zhu, Ya-lin A. Huang, Ashley Townes, Kevin P. Delaney, Karen W. Hoover

**Affiliations:** ^1^Division of HIV Prevention, National Center for HIV/AIDS, Viral Hepatitis, STD, and TB Prevention, CDC; ^2^Oak Ridge Institute for Science and Education, Oak Ridge, Tennessee.

HIV testing is a critical component of effective HIV prevention and care. CDC recommends routine opt-out HIV testing in health care settings for all sexually active persons aged 13–64 years at least once in their lifetime and risk-based testing regardless of age for those who report behaviors associated with HIV acquisition (*1*). However, recent studies show low HIV testing rates in clinical settings; HIV testing rates at visits to physician offices did not increase during 2009–2016 (*2*). The objective of the current study is to estimate temporal trends in HIV testing among persons with commercial insurance or Medicaid from 2014 through 2019 and describe their demographic characteristics in 2019. Weighted data from the IBM MarketScan Commercial Claims and Encounters database* (commercial insurance) and from the Centers for Medicare & Medicaid Services (CMS) claims database^†^ (Medicaid) were analyzed to estimate the proportions of persons with commercial insurance or Medicaid who received testing for HIV. Testing rates increased among male and nonpregnant female persons aged ≥13 years with either type of coverage. In 2019, only 4.0% of those with commercial insurance and 5.5% of those with Medicaid received testing for HIV. Testing rates were higher among non-Hispanic Black or African American (Black) persons and Hispanic or Latino (Hispanic) persons. Based on mathematical modeling studies, these annual testing rates would need to increase at least threefold and be sustained over several years (*3*,*4*) to achieve the Ending the HIV Epidemic (EHE) in the U.S. initiative goal of ≥95% of persons with HIV being aware of their infection by 2025.^§^ Interventions need to be implemented to increase routine and risk-based HIV testing in clinical settings to higher levels that can help reduce disparities in HIV diagnoses between Black and Hispanic persons compared with non-Hispanic White (White) persons (*5*). Increased HIV testing is essential to achieve the goals of the EHE initiative and reduce disparities in HIV diagnoses; public health should partner with health care systems to implement interventions that support increased testing.

Many factors might be associated with low HIV testing rates for persons across socioeconomic strata, even among those with health care insurance (*6*). In 2019, the U.S. Department of Health and Human Services launched the EHE initiative that includes four strategic pillars (diagnose, treat, prevent, and respond) to end the HIV epidemic by 2030. The “diagnose” pillar is intended to achieve diagnosis for all persons with HIV as early as possible, with a goal to detect ≥95% of all infections by 2025. As part of the initiative, CDC funded health departments to conduct several activities, including the expansion of routine and risk-based testing in clinical settings.^¶^ HIV testing can serve as an entry point for HIV prevention and care services and can normalize HIV testing as a routine part of preventive care.

CDC analyzed data from the 2014–2019 MarketScan and Medicaid databases to identify temporal trends in HIV testing in clinical settings among persons with commercial insurance or Medicaid and their demographic characteristics. The MarketScan database is a convenience sample of commercial health plans that include health service information for approximately 40 million persons per year and is weighted using validated methods to be nationally representative of the 200 million U.S. persons with commercial insurance. The CMS database includes information on persons with Medicaid in all 50 states and the District of Columbia. Both databases contained deidentified patient information and diagnostic, procedural, and drug codes for clinical services provided; Medicaid reports data on race/ethnicity, and MarketScan does not. Separate analyses were conducted using the MarketScan and Medicaid databases. Eligibility criteria included persons who 1) were aged ≥13 years, 2) were continuously enrolled for at least 6 months in a given year, and 3) had no previous HIV diagnosis. Pregnant adolescents and women were excluded because CDC recommends that they receive prenatal testing for HIV during each pregnancy, rather than routine or risk-based testing (*1*). Persons aged ≥65 years were included because HIV prevalence has increased in the oldest age group for which surveillance data are reported (*7*). HIV diagnoses were identified using codes from the ninth and tenth revisions of the International Classification of Diseases.** HIV tests were identified using Current Procedural Terminology^††^ and Healthcare Common Procedure Coding System^§§^ codes. SAS software (version 9.4; SAS Institute) was used to conduct analyses. This activity was reviewed by CDC and was conducted consistent with applicable federal law and CDC policy.^¶¶^

The proportions of male and nonpregnant female persons aged ≥13 years with commercial insurance or Medicaid who received HIV testing each year were estimated. Race/ethnicity data were available only for persons with Medicaid, therefore the trend in testing over time was estimated by race/ethnicity only for those with Medicaid. The estimated annual percentage change and 95% confidence intervals were calculated for each trend. The estimated number and proportion of persons with commercial insurance and with Medicaid who had testing in 2019 were stratified by sex, age group, race/ethnicity (Medicaid only), urban versus rural residence, and U.S. Census region.

During 2014–2019, the proportion of male and nonpregnant female persons aged ≥13 years with HIV testing increased an estimated 6.0% per year among those with commercial insurance, and an estimated 3.2% among those with Medicaid (Table 1). Among persons with Medicaid, this trend was observed for all racial and ethnic groups except Hispanic persons (Figure). Despite the increase in HIV testing, only 4.0% of persons with commercial insurance and 5.5% of persons with Medicaid received testing for HIV in 2019 (Table 1). The proportion of persons with HIV testing was higher among those with Medicaid than among those with commercial insurance across all regions and all demographic groups except persons aged ≥65 years (Table 2). In 2019, among persons with Medicaid, the percentages of Black persons (8.5%) and Hispanic persons (5.9%) with HIV testing were higher than the percentages of White persons (3.9%) and non-Hispanic Asian (Asian) persons (5.0%) with HIV testing.

**TABLE 1:** Number and percentage of male and nonpregnant female persons aged ≥13 years who received testing for HIV and the estimated annual percentage change in HIV testing among persons with commercial insurance or Medicaid — United States, 2014–2019

Insurance type/Insured persons	Year						EAPC* (95% CI)
	2014	2015	2016	2017	2018	2019	
**Commercial^†^**							
Unweighted no.	32,965,590	19,983,855	19,897,709	18,747,383	19,122,236	17,471,826	N/A
Weighted no.^§^	110,689,206	117,747,637	112,914,294	115,710,035	114,177,141	114,726,222	N/A
Weighted no. with HIV test^§^	3,486,360	3,540,501	3,408,869	3,781,412	4,247,939	4,637,964	6.4 (6.3–6.4)
Weighted % with HIV test	3.1	3.0	3.0	3.3	3.7	4.0	6.0 (6.0–6.1)
**Medicaid^†^**							
**Total no.**	**45,964,636**	**51,684,583**	**52,911,975**	**53,444,150**	**53,126,192**	**52,472,143**	**N/A**
No. with HIV test	2,284,238	2,371,188	2,606,385	2,794,386	2,844,232	2,898,425	5.2 (5.2–5.2)
% with HIV test	5.0	4.6	4.9	5.2	5.4	5.5	3.2 (3.1–3.2)

**Abbreviations:** CI  =  confidence interval; EAPC = estimated annual percentage change; N/A = not applicable.

* EAPCs were calculated using a generalized Poisson model.

**^†^** Persons who were continuously insured for at least 6 months in a given year.

^§^ Weighted to generate nationally representative estimates of persons with commercial insurance (https://www.ibm.com/products/marketscan-research-databases/databases).

**FIGURE:**
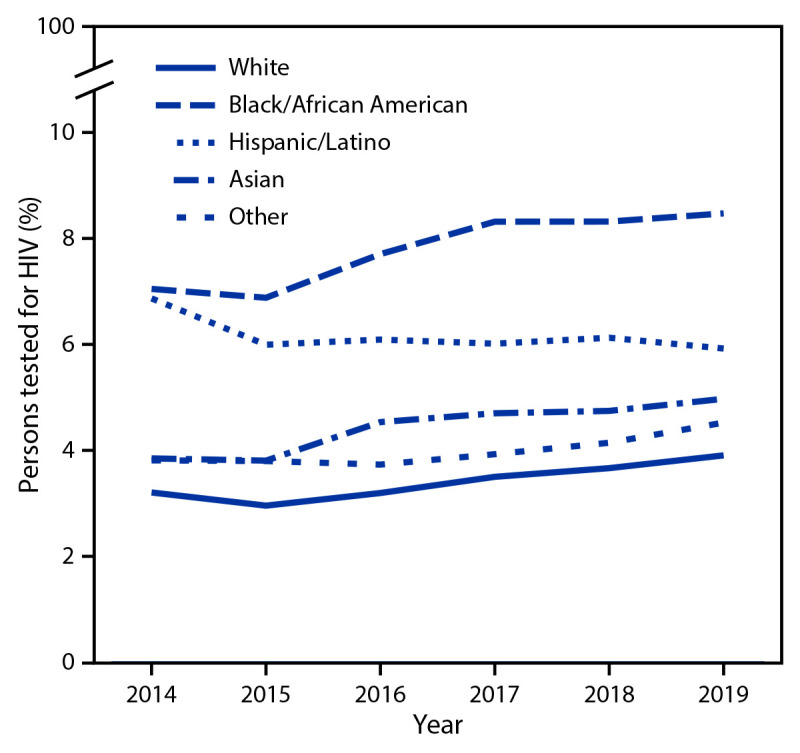
Percentage of male and nonpregnant female persons aged ≥13 years with Medicaid who **r**eceived testing for HIV, by race and ethnicity* — Centers for Medicare & Medicaid Services, United States, 2014–2019 * Persons reported as White, Black, Asian, and Other were non-Hispanic; persons reported as Hispanic/Latino could be of any race.

**TABLE 2:** Number and percentage of male and nonpregnant female persons aged ≥13 years with commercial insurance or Medicaid who received testing for HIV, by demographic characteristics — United States, 2019

Characteristic	Insured persons*				
	Commercial insurance			Medicaid	
	Unweighted no.	Weighted no.^†^	Weighted no. with HIV test^†^ (%)	No.	No. with HIV test (%)
**Total**	**17,471,826**	**114,726,222**	**4,637,964 (4.0)**	**52,472,143**	**2,898,425 (5.5)**
**Sex at birth**					
Male	8,545,670	57,671,191	2,129,687 (3.7)	22,869,597	1,084,432 (4.7)
Female	8,926,156	57,055,031	2,508,276 (4.4)	29,602,546	1,813,993 (6.1)
**Age group, yrs**					
13–14	614,706	4,137,555	17,427 (0.4)	3,550,836	34,672 (1.0)
15–18	1,144,235	7,924,306	173,427 (2.2)	6,272,678	279,257 (4.5)
19–29	3,600,398	22,265,021	1,515,024 (6.8)	10,011,887	855,013 (8.5)
30–49	6,650,691	45,192,213	2,103,739 (4.7)	14,950,792	1,140,752 (7.6)
50–64	5,448,381	35,119,257	827,531 (2.4)	9,816,896	529,234 (5.4)
≥65	13,415	87,870	816 (0.9)	7,869,054	59,497 (0.8)
**Race/Ethnicity**					
White	—^§^	—	—	19,713,421	769,135 (3.9)
Black/African American	—	—	—	9,283,337	785,673 (8.5)
Hispanic/Latino^¶^	—	—	—	11,379,127	673,073 (5.9)
Asian	—	—	—	2,636,311	130,950 (5.0)
Other**	—	—	—	1,012,462	45,751 (4.5)
Unknown	—	—	—	8,447,485	493,843 (5.8)
**Urban/Rural residence^††^**					
Urban	13,853,880	94,995,029	4,195,184 (4.4)	41,294,013	2,503,400 (6.1)
Rural	1,867,957	11,658,371	204,827 (1.8)	9,747,177	292,824 (3.0)
Unknown	1,749,989	8,072,823	237,952 (2.9)	1,430,953	102,201 (7.1)
**U.S. Census region^§§^**					
Northeast	3,199,361	20,951,646	1,204,664 (5.7)	10,228,160	732,846 (7.2)
Midwest	3,492,100	25,925,386	707,469 (2.7)	9,274,664	468,835 (5.1)
South	7,916,680	41,356,545	1,646,199 (4.0)	15,431,589	710,537 (4.6)
West	2,801,976	26,430,883	1,076,942 (4.1)	16,444,186	923,992 (5.6)
Unknown	61,709	61,762	2,690 (4.4)	1,094,450	62,222 (5.7)

* Persons who were continuously enrolled in their health insurance plan for ≥6 months in 2019.

**^†^** Weighted to generate nationally representative estimates of persons with commercial insurance (https://www.ibm.com/products/marketscan-research-databases/databases).

^§^ Dashes indicate race/ethnicity data not available in commercial insurance data set.

^¶^ Race/ethnicity groups are mutually exclusive. Hispanic/Latino persons can be of any race.

** “Other” includes American Indian or Alaska Native and Native Hawaiian or Other Pacific Islander.

^††^ Location of patient residence. For persons with commercial insurance, their urban or rural residence was defined using Metropolitan Statistical Areas codes. For persons with Medicaid, their urban or rural location was defined using their zip code and the Centers for Medicare & Medicaid Services carriers’ Medicare Administrative Contractors and localities files (https://www.cms.gov/Medicare/Medicare-Fee-for-Service-Payment/FeeScheduleGenInfo).

^§§^ *Northeast:* Connecticut, Maine, Massachusetts, New Hampshire, New Jersey, New York, Pennsylvania, Rhode Island, and Vermont. *Midwest:* Illinois, Indiana, Iowa, Kansas, Michigan, Minnesota, Missouri, Nebraska, North Dakota, Ohio, South Dakota, and Wisconsin. *South:* Alabama, Arkansas, Delaware, District of Columbia, Florida, Georgia, Kentucky, Louisiana, Maryland, Mississippi, North Carolina, Oklahoma, South Carolina, Tennessee, Texas, Virginia, and West Virginia. *West:* Alaska, Arizona, California, Colorado, Hawaii, Idaho, Montana, Nevada, New Mexico, Oregon, Utah, Washington, and Wyoming.

## Discussion

HIV testing rates increased from 2014 through 2019 among persons with commercial insurance and persons with Medicaid. The proportion of persons who received HIV testing was higher among those with Medicaid than among those with commercial insurance; trends were generally similar across demographic characteristics.

Higher rates of HIV testing were expected among persons with Medicaid because Medicaid includes large proportions of persons in populations with the highest rates of HIV diagnoses. A recent study found that from 2009 to 2014 HIV testing increased in community health centers, and this trend likely continued during the period of this study (*2*). Guidelines for routine opt-out and risk-based HIV testing have been widely disseminated, and testing campaigns have been conducted by public health organizations and health care systems to increase provider awareness of these recommendations. Testing also might have increased as HIV preexposure prophylaxis (PrEP) use increased during the same period because PrEP users should receive HIV testing at PrEP initiation and every 3 months thereafter (*7*,*8*). HIV testing is a strategic priority of EHE and was included among Medicaid noncore health care quality measures for adults in 2021 (*9*), which could contribute to future increases in HIV testing rates.

The findings in this report are subject to at least four limitations. First, only persons with 6 months of continuous commercial insurance or Medicaid enrollment were included, which might have resulted in an underestimate or overestimate of testing rates. Length of enrollment might vary by a person’s demographic characteristics and result in under- or overestimation of HIV testing rates by these characteristics. Second, because there was no link between persons included in the MarketScan and Medicaid databases, accounting for persons enrolled in both Medicaid and commercial insurance in the same year was not possible. This circumstance might have resulted in counting a person as having been tested in both the commercial insurance and Medicaid analyses in the same year. However, by limiting the analyses to persons enrolled in their health plan for at least 6 months, it is unlikely that many such persons were included in analyses for both systems. Third, Medicare recipients who were not dually enrolled in Medicaid or commercial insurance were not included, so this study included only limited HIV testing information for persons aged ≥65 years. Finally, persons receiving testing at an HIV outreach event or in a venue that did not bill a person’s health insurance for the HIV test would not have been included in this study.

HIV testing rates were highest among Black persons and Hispanic persons, which is encouraging. To accomplish goals of the EHE initiative and to reduce disparities in HIV diagnoses, higher HIV testing rates are needed for all groups, but especially for some racial and ethnic minority groups (*4*). A recent study found that a two- to threefold increase in HIV testing rates at ambulatory care visits would result in almost all Black men and Hispanic men receiving testing by age 39 years (*3*). In another recent study, a standing order for a routine opt-out HIV test added to all blood draws in a large health care system in 2016 resulted in 35.4% of the patient population receiving an HIV test (*10*). These percentages were much higher than the national percentages found in the current study (4.0% among persons with commercial insurance and 5.5% among those with Medicaid). A recent review conducted by the Community Preventive Services Task Force (CPSTF) found that testing can be efficiently increased at clinical visits by incorporating clinical decision support tools in electronic health records that generate an automated order for routine opt-out testing or risk-based testing (*5*). Increased HIV testing in clinical settings is essential to achieve the goals of the EHE initiative and reduce disparities in HIV diagnoses. Public health should partner with health care systems to implement interventions, such as those reviewed by the CPSTF, that support increased testing.

**Summary**

**What is already known about this topic?**

HIV testing in clinical settings provides an entry point for other HIV care and prevention services.

**What is added by this report?**

The percentage of males and nonpregnant females aged ≥13 years with either commercial insurance or Medicaid who received testing for HIV increased from 2014 to 2019 but was less than 6%. Testing rates were higher among persons with Medicaid than those with commercial insurance; among those with Medicaid, rates for Black/African Americans and Hispanic/Latino persons were higher than for White persons.

**What are implications for public health practice?**

Increasing HIV testing in clinical settings by at least threefold is essential to achieve the goal of ≥95% of persons with HIV being aware of their infection by 2025 and to reduce disparities in HIV diagnoses.

## Footnotes

*
https://www.ibm.com/products/marketscan-research-databases/databases

† https://www.cms.gov/Research-Statistics-Data-and-Systems/Computer-Data-and-Systems/MedicaidDataSourcesGenInfo. Access to this database is by license only.

§
https://www.hiv.gov/federal-response/ending-the-hiv-epidemic/overview

¶
https://www.cdc.gov/hiv/funding/announcements/ps20-2010/index.html

** *International Classifications of Diseases, Ninth Revision (*ICD-9) diagnosis codes of 042, 079.53, 795.71 were used to identify persons with an HIV diagnosis. https://www.cdc.gov/nchs/icd/icd9.htm. *International Classifications of Diseases, Tenth Revision (*ICD-10 diagnosis codes of B20, B97.35, O98.7XX, R75, V08, Z21 were used to identify persons with an HIV diagnosis. https://www.cdc.gov/nchs/icd/icd10.htm

†† Current Procedural Terminology codes 86689, 86701–86703, and 87389–87391 were used to identify HIV testing procedures. https://www.ama-assn.org/practice-management/cpt

§§ Healthcare Common Procedure Coding System codes G0432-G0435 were also included to identify HIV testing. https://www.cms.gov/Medicare/Coding/MedHCPCSGenInfo

¶¶ 45 C.F.R. part 46, 21 C.F.R. part 56; 42 U.S.C. Sect. 241(d); 5 U.S.C. Sect. 552a; 44 U.S.C. Sect. 3501 et seq.
